# Dose response relationship between food insecurity and quality of life in United States adults: 2016–2017

**DOI:** 10.1186/s12955-023-02103-3

**Published:** 2023-03-08

**Authors:** Sanjay Bhandari, Jennifer A. Campbell, Rebekah J. Walker, Abigail Thorgerson, Aprill Z. Dawson, Leonard E. Egede

**Affiliations:** 1 Department of Medicine, Division of General Internal Medicine, Medical College of Wisconsin, 8701 Watertown Plank Rd., Milwaukee, WI 53226 USA; 2 Center for Advancing Population Science, Medical College of Wisconsin, 8701 Watertown Plank Rd, Milwaukee, WI 53226 USA

## Abstract

**Background:**

Food insecurity is associated with worse general health rating, but little research exists investigating whether there is a dose response relationship across levels of food security and mental and physical health domains at the population level.

**Methods:**

Data from the Medical Expenditure Panel Survey (2016–2017) with US adults aged 18 years and older was used. The physical component score (PCS) and mental component score (MCS) of Quality of Life, served as the outcome measures. Four categories of food insecurity (high, marginal, low, very low food security) served as the primary independent variable. Linear regression was used to run unadjusted followed by adjusted models. Separate models were run for PCS and MCS.

**Results:**

In a sample of US adults, 16.1% reported some degree of food insecurity. For PCS, marginal (β = − 2.54 (*p* < 0.001), low (β = − 3.41, (*p* < 0.001), and very low (β = − 5.62, (*p* < 0.001) food security was associated with worse PCS scores, compared to adults with high food security. For MCS, marginal (β = − 3.90 (*p* < 0.001), low (β = − 4.79, (*p* < 0.001), and very low (β = − 9.72, (*p* < 0.001) food security was associated with worse MCS scores, compared to adults with high food security.

**Conclusion:**

Increasing levels of food insecurity were associated with decreased physical and mental health quality of life scores. This relationship was not explained by demographic factors, socioeconomic factors, insurance, or comorbidity burden. This study suggests work is needed to mitigate the impact of social risk, such as food insecurity, on quality of life in adults, and understand pathways and mechanisms for this relationship.

## Introduction

Food insecurity is defined as the limited or uncertain access to, or ability to acquire, nutritionally adequate food (USDA). In 2020, over 38 million or 11.8% of US adults lived in food insecure households, with 8% reporting low food security and 3.8% reporting very low food security [1]. Evidence shows food insecurity is strongly associated with a range of health conditions, such as hypertension, coronary artery disease, diabetes, hepatitis, stroke, cancer, asthma, arthritis, chronic obstructive pulmonary disease, kidney disease, depression, and limitations in activities [2–5]. Food insecure individuals have higher rates of emergency department visits and hospitalizations even after accounting for other socioeconomic factors [6]. Further, food insecurity poses a substantial financial burden on society accounting for an annual $77.5 billion in additional health care expenditure [6, 7].

Given its prevalence, associated adverse health outcomes, and increased healthcare costs, growing attention towards addressing food insecurity as a social need is taking place across several professional medical societies, health insurance payers, and healthcare organizations who have invested in and recommend screening for food insecurity with referral to food resources [8–12]. While such attention is serving to grow the evidence base for how to address food insecurity at a population level, there remain gaps in understanding the impact that food insecurity has on health status at the population level. Specifically, there is need to further understand the relationship between food insecurity and patient reported outcomes, namely health related quality of life (HRQoL), individual perception of one’s physical and mental health status as well the factors that influence their functioning [13], to further inform food insecurity interventions from a holistic standpoint.

Although various studies have highlighted the inverse relationship between food insecurity and HRQoL in general [14–21], there is limited knowledge on whether a dose–response relationship exists between food insecurity and HRQoL and whether the relationship is consistent across both physical and mental health domains of HRQoL at the population level. Therefore, this paper aims to address this gap by examining the dose–response relationship between food insecurity and HRQoL using a nationally representative sample of US adults. We hypothesize that worsening levels of food insecurity will be associated with worsening HRQoL and this dose–response relationship will be consistent across both physical and mental domains of HRQoL.

## Methods

### Data source

The 2016 and 2017 Medical Expenditure Panel Survey (MEPS) was analyzed as a cross-sectional analysis. These two years included detailed questions on food insecurity and included measures of HRQOL. The full year consolidated files and food security files from the household component files were used. MEPS is a national survey that collects data from U.S. citizens and their families on items such as health services, employment, and insurance. This survey began recording in 1996 and is overseen by the Agency for Healthcare Research and Quality (AHRQ) [22]. The sample included adults 18 and older who serve as the reference person in the food insecurity file.

### Measures

#### Quality of life

The primary outcomes are physical component summary (PCS) and mental component summary (MCS), both of which are continuous measures. The MEPS Self-Administered Questionnaire uses the Short Form-12 Health Survey Version 2 forming two summary scores based on responses to these questions for PCS and MCS. Both MCS and PCS assess four key domains, respectively. For MCS these include: (1) vitality; (2) social functioning; (3) role emotional; and (4) mental health. For PCS these include: (1) functioning; (2) role physical; (3) bodily pain; and (4) general self-rated health. Example questions include: “How much of the time during the past 4 weeks have you felt calm and peaceful?”, and “During the past 4 weeks, how much did pain interfere with your normal work?”. Information from all 12 questions were used as part of the scoring algorithms for both the PCS and the MCS, which was completed by AHRQ [22]. Both scales are scored based on an adult population mean of 50, with a standard deviation of 10. In both scales higher scores represents higher functioning [22].

#### Food insecurity

The primary independent variable is household food security. This variable is based on the USDA 10-item Adult Food Security Scale and previous literature [23, 24]. The raw score was created by adding the affirmative answers to the following prompts:

1. “How often in the last 30 days has anyone in the household worried whether food would run out before getting money to buy more?” Score of 1 if responded ‘often’ or ‘sometimes’.
2. “How often in the last 30 days did the food purchased not last and the person/household didn't have money to get more?” Score of 1 if responded ‘often’ or ‘sometimes’.
3. “How often in the last 30 days could the person/household not afford to eat balanced meals?” Score of 1 if responded ‘often’ or ‘sometimes’.
4. “In the last 30 days, did the person/household reduce or skip meals because there wasn't enough money for food?” Score of 1 if responded ‘yes’.
5. “How many meals were skipped in the last 30 days?” Score of 1 if responded with 3 + days.
6. “In the last 30 days, did the person/household ever eat less because there wasn't enough money for food?” Score of 1 if responded ‘yes’.
7. “In the last 30 days, was the person/household ever hungry but didn't eat because there wasn't enough money for food?” Score of 1 if responded ‘yes’.
8. “In the last 30 days, did anyone in the household lose weight because there wasn't enough money for food?” Score of 1 if responded ‘yes’.
9. “In the last 30 days, did anyone in the household not eat for a whole day because there wasn't enough money for food?” Score of 1 if responded ‘yes’.
10. “How many days in the last 30 days did anyone in the household not eat for a whole day because there wasn't enough money for food?” Score of 1 if responded with 3 + days.

The score was adjusted so that those who answered 3 + days to, “how many meals were skipped in the last 30 days?” as well as to, “how many days in the last 30 days did anyone in the household not eat for a whole day because there wasn't enough money for food?” had an additional 1 added to their overall score. This addition is based on Dean et al. [23] to provide comparability with the USDA Economic Research Service guidelines by aligning the 30-day window in MEPS questions to the 12-month window in USDA questions [23]. The intent of this scoring is to appropriately capture the severity of food insecurity when skipping meals or not eating for an entire day occurs multiple times. Missing was defined as refusing to answer, responding with “I don’t know” or “Not ascertained” to all food insecurity questions. If an individual answered at least one of the questions, they had a score calculated using the items with a response in the dataset.

The raw score was grouped into 4 categories: high food security (score of 0), marginal food security (score of 1–2), low food security (score of 3–5), and very low food security (score of 6–11).

#### Covariates

The covariates included age (18–44, 45–64, 65 +), sex (male, female),race/ethnicity (Non-Hispanic White, Non-Hispanic Black, Hispanic, other), education (less than high school, high school graduate, college or more), employment (employed, not employed), region (Midwest, Northwest, South, West), poverty (poor/near poor: ≤ 124% poverty line, low income: 125–199% poverty line, middle income: 200–399% poverty line, high income: ≥ 400% poverty line), and insurance (private, public, uninsured).

### Statistical analyses

Population estimates were created using sampling weights as recommended by MEPS. Sample demographics were reported as percentages and/or mean with standard deviation. Primary outcomes were PCS and MCS scores treated as continuous variables, while the primary independent variable for each model was the 4 category food security variable with full food security as the reference group. Covariates included age, sex, race/ethnicity, education, region, poverty level, insurance status, and employment status. Initial models included unadjusted linear regression for the independent relationship between food security and HRQOL (PCS and MCS respectively). Then, fully adjusted linear regression models were run with PCS and MCS as separate outcome variables, the 4-category food security variable as the primary independent variable, and age, sex, race/ethnicity, education, region, poverty level, insurance status, and employment status as covariates. Covariates were included based on conceptual relevance and/or bivariate *p*-values < 0.25. All analyses were done in R package (Version 4.0.3). A *p*-value less than 0.05 was used to determine statistical significance.

## Results

The total sample included 26,196 adult participants with food security data, representing 275,829,365 non-institutionalized United States adults aged 18 years and older. The sample characteristics are given in Table 1 with 83.9% of participants indicating full food security, 6.6% having marginal food security, 4.9% having low food security, and 4.6% having very low food security.

**Table 1:** Sample characteristics of United States adults: 2016–2017

Characteristics	Total sample n = 26,196 (weighted %)
*Age*	
18–44	40.3
45–64	35.8
65 +	23.9
*Sex*	
Male	47.5
Female	52.5
*Race/ethnicity*	
Non-hispanic white	65.4
Non-hispanic black	12.5
Hispanic	13.8
Other	8.3
*Education*	
Less than high school	10.8
High school grad	27.1
College or more	61.7
*Employment*	
Employed	64.0
Not employed	35.9
*Region*	
Midwest	21.4
Northeast	17.5
South	37.8
West	23.2
*Poverty*	
Poor/near poor	17.8
Low income	13.1
Middle income	28.6
High income	40.5
*Insurance*	
Private	69.4
Public	23.1
Uninsured	7.5
*Food insecurity*	
High food security	83.9
Marginal food security	6.6
Low food security	4.9
Very low food security	4.6
*Quality of life*	
Physical component	49.22 (range: 4.41–74.07)
Mental component	51.79 (range 0.04–75.64)

The unadjusted and adjusted linear regression models for PCS are shown in Table 2. In the unadjusted model, those with marginal food security had a 2.91 decrease in PCS, on average, compared to those with full food security (β = − 2.91, 95% CI [− 3.65, − 2.16], *p*-value < 0.001). Those with low food security had a 4.45 decrease (β = − 4.45, 95% CI [− 5.20, − 3.69], *p*-value < 0.001), on average, and those with very low food security had a 7.29 decrease, on average (β = − 7.29, 95% CI [− 8.24, − 6.34], *p*-values < 0.001). When adjusted by age, sex, race/ethnicity, education, region, poverty level, insurance status, and employment status, those with marginal food security had a 2.54 decrease in PCS, on average, compared to those with full food security (β = − 2.54, 95% CI [− 3.14, − 1.94], *p*-value < 0.001). Those with low food security had a 3.41 decrease, on average, compared to those with full food security (β = − 3.41, 95% CI [− 4.03, − 2.80], *p*-value < 0.001) and those with very low food security had a 5.62 decrease, on average, compared to those with full food security (β = − 5.62, 95% CI [− 6.36, − 4.85], *p*-values < 0.001).

**Table 2:** Unadjusted and Adjusted Linear Regression Model Examining the Association between Food Insecurity and PCS, United States Adults: 2016–2017

Food security category	PCS quality of Life	
	Unadjusted	Adjusted
	β (CI)	β (CI)
Marginal food security	− 2.91*** (− 3.65; − 2.16)	− 2.54*** (− 3.14; − 1.94)
Low food security	− 4.45*** (− 5.20; − 3.69)	− 3.41*** (− 4.03; − 2.80)
Very low food security	− 7.29*** (− 8.24; − 6.34)	− 5.62*** (− 6.39; − 4.85)

Ref: High Food Security. *p < 0.05; **p < 0.01; ***p < 0.001. Adjusted for age, sex, race/ethnicity, education, employment, region, poverty level, insurance status

The unadjusted and adjusted linear regression models for MCS are shown in Table 3. In the unadjusted model, those with marginal food security had a 4.70 decrease in MCS, on average, compared to those with full food security (β = − 4.70, 95% CI [− 5.33, − 4.07], *p*-value < 0.001). Those with low food security had a 5.81 decrease, on average, compared to those with full food security (β = − 5.81, 95% CI [− 6.43, − 5.18], *p*-value < 0.001); and those with very low food security had a 11.24 decrease, on average, compared to those with full food security (β = − 11.24, 95% CI [− 12.12, − 10.37], (*p*-values < 0.001). When adjusted by age, sex, race/ethnicity, education, region, poverty level, insurance status, and employment status, those with marginal food security had a 3.90 decrease in MCS, on average, compared to those with full food security (β = − 3.90, 95% CI [− 4.53, − 3.28], *p*-value < 0.001). Those with low food security had a 4.79 decrease, on average, compared to those with full food security, (β = − 4.79, 95% [− 5.45, − 4.12], *p*-value < 0.001); and those with very low food security had a 9.72 decrease, on average, compared to those with full food security (β = − 9.72, 95% CI [− 10.62, − 8.82], (*p*-values < 0.001).

**Table 3:** Unadjusted and adjusted linear regression model examining the association between food insecurity and MCS, United States adults: 2016–2017

Food security category	MCS quality of Life	
	Unadjusted	Adjusted
	β (CI)	β (CI)
Marginal food security	− 4.70*** (− 5.33; − 4.07)	− 3.90*** (− 4.53; − 3.28)
Low food security	− 5.81*** (− 6.43; − 5.18)	− 4.79*** (− 5.45; − 4.12)
Very low food security	− 11.24*** (− 12.12; − 10.37)	− 9.72*** (− 10.62; − 8.82)

Ref: High Food Security. *p < 0.05; **p < 0.01; ***p < 0.001. Adjusted for age, sex, race/ethnicity, education, employment, region, poverty level, insurance status

## Discussion

Overall, in a nationally representative sample of approximately 2.8 million US adults, this study shows that 16.1% of the US population reported experiencing marginal, low, or very low food security. Increasing levels of food insecurity were found to be associated with worsening physical and mental HRQoL scores, consistent with our hypotheses. Specifically, for physical health related quality of life, after adjusting for demographic factors, marginal food security was significantly associated with 2.54 lower points, low food security with 3.41 lower points, and very low food security with 5.62 lower points, compared to adults with full food security. For mental health related quality of life, marginal food security was significantly associated with 3.90 lower points, low food security was associated with 4.79 lower points, and very low food security was associated with 9.72 lower points compared to adults with full food security.

These findings add to our current understanding by demonstrating at a population level that a dose–response relationship exists between increasing levels of food insecurity and both physical and mental health related quality of life. While few studies have examined the dose–response relationship between food insecurity and HRQoL at a population level, these findings are consistent with the current literature. For example, existing evidence shows the relationship between food insecurity and mental health outcomes, underscoring the presence of worse mental health outcomes, including depression, in food insecure individuals [17, 19, 21]. However, many of these studies have been limited in generalizability by focusing on specific sub-populations with chronic conditions and lacking national representation. Further, many studies have used inconsistent measures of quality of life. For example, two recent studies used nationally representative samples of the US population and evaluated the association between food insecurity and quality of life using various validated patient-reported outcome scales [25, 26]. While both studies highlight the negative association between food insecurity and HRQoL, the study by Hammer et al. specifically showed variability of the association based on the scales used, with the recommended measure being the abbreviated derivative of the present study’s measure of HRQoL.

In addition, the current findings show that marginal food security is significantly associated with lower HRQoL compared to those with full food security. This is a notable finding as marginal food security and full food security are often collapsed into one food secure category [3]. Consistent with existing evidence showing that marginal food security is related to worse health outcomes across the life span for health and well-being [27–29], these findings add to the current body of literature highlighting the need to examine food insecurity across categories, rather than collapsing into a binary indicator. The implications of our study findings are multifold. Food insecurity is included as one of the five core domains of the Centers for Medicare & Medicaid Services (CMS)’s Accountable Health Communities Health-Related Social Needs Screening Tool.(The Centers for Medicare & Medicaid Services) The Supplemental Nutrition Assistance Program (SNAP), the nation’s largest federally funded food program to counteract food insecurity, serves approximately 1 in 7 Americans; enrollment in SNAP has been shown to be linked with improved outcomes across various dimensions, including improvement in food insecurity measures, reduction in mortality and poverty, lower healthcare costs, and improved medication adherence [30–34] Given the strong inverse relationship of food insecurity and HRQoL, it is critical to incorporate HRQoL in the current food interventions and public health initiatives to effectively address food insecurity from a holistic standpoint.

Owing to HRQoL’s role as a predictor for treatment success, and as an important patient reported outcome in medical decision-making and evaluation of practices and policies, the number of research studies reporting on HRQoL has steadily increased in the past decades [35–37]. Evidence shows worse HRQoL is associated with increased mortality and healthcare utilization across various groups of patient populations [38–40]. Therefore, it is critical to inform interventions that will reduce rates of food insecurity as a way to improve HRQoL. More specifically, given the current study’s findings demonstrating the dose–response relationship between food insecurity with HRQoL, future research should focus on reversing the major decline in HRQoL across all gradients and identify whether the effect will differ by the duration or modalities of intervention (directly providing food, food vouchers, monetary assistance, etc.).

Additionally, as availability of data on HRQoL will help researchers to further evaluate the relationship between food insecurity and HRQoL, the current findings can inform efforts to better evaluate HRQoL at the population level by health insurance payers encouraging collection of data through value-based payment models in addition to addressing food insecurity. Existing food programs such as SNAP can also consider routinely collecting HRQoL data from its participants to substantiate robust effectiveness of the program, which can also serve as a valuable information for federal government/Congress regarding SNAP program.

Finally, given the findings that the dose response relationship between food security categories and HRQOL are independent of sociodemographic factors and comorbidity, it will be important to understand the potential pathways and mechanisms of these effects. Longitudinal studies and studies that use path analysis or structural equation modeling may be warranted to better understand these relationships and identify potential targets for intervention.

Although there are several strengths of our study including a nationally representative sample and incorporation of both physical and mental health components of HRQoL measure, a few limitations exist. First, given the cross-sectional nature of the study, causality between food insecurity (exposure variable) and HRQoL (outcome) cannot be inferred. Second, recall biases cannot be ruled out as the data are based on self-report. Finally, there might be some residual confounders that were not accounted for.

## Conclusion

In conclusion, our study showed that increasing levels of food insecurity were associated with worsening physical and mental health related quality of life scores in a dose–response relationship. Specifically, this study found that marginal food security was significantly associated with worse HRQoL compared to full food security. This relationship across categories of food security was not explained by demographic factors, socioeconomic factors, or insurance status. Since HRQoL is an important patient reported outcome and is associated with various clinical outcomes, further research is needed to mitigate the impact of food insecurity on HRQoL in adults. Specifically, next steps in the field should emphasize identification of potential pathways and mechanisms of the relationship as well as development of interventions to reduce rates of food insecurity as a way to improve HRQoL across both physical and mental health domains.

## Data Availability

The datasets during and/or analyzed during the current study available from the corresponding author on reasonable request.
